# Supplementary material for: Aggregatibacter actinomycetemcomitans LtxA Hijacks Endocytic Trafficking Pathways in Human Lymphocytes
Source: Pathogens. 2020 Jan 21;9(2):74. doi: 10.3390/pathogens9020074 (PMC7168647; doi:10.3390/pathogens9020074)
Supplement: Supplementary file 1 [file pathogens-09-00074-s001.pdf]

pathogens

MDPI

Supplementary Data

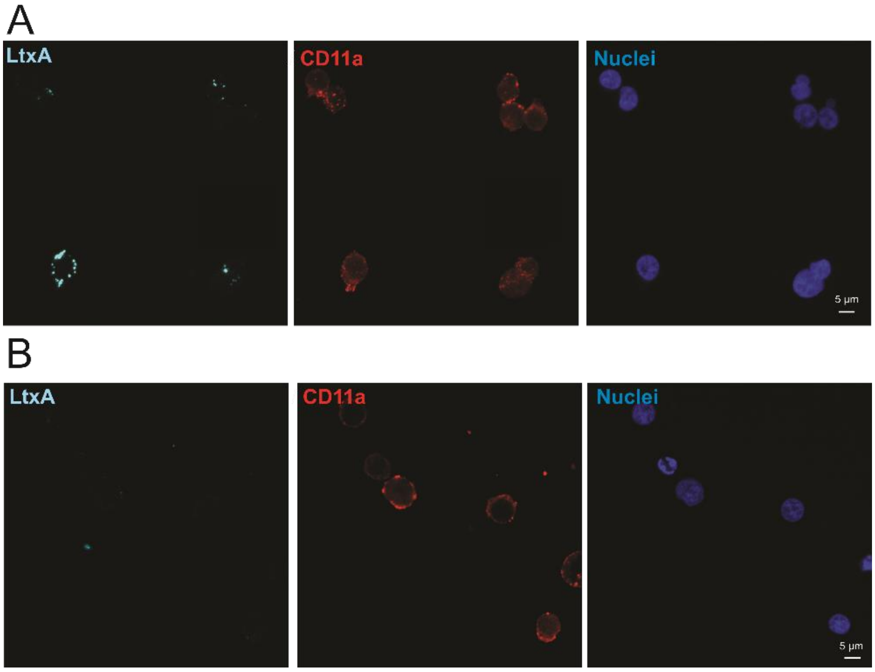

Figure S1. Confocal imaging of LtxA and CD11a in Jn.9 cells. Jn.9 cells were treated with 20 nM LtxA-DY650 for 30 min at 37 °C (A) and at 4 °C (B). Immunostaining and confocal imaging were performed as described in the "*Immunofluorescence*" section of "Materials and Methods". LtxA-DY650 is shown in cyan. CD11a, recognized with anti-human CD11a antibody (clone HI111) conjugated with Alexa Fluor™ 594, is shown in red. Nuclei stained with Hoechst dye are shown in blue. Representative images are shown.

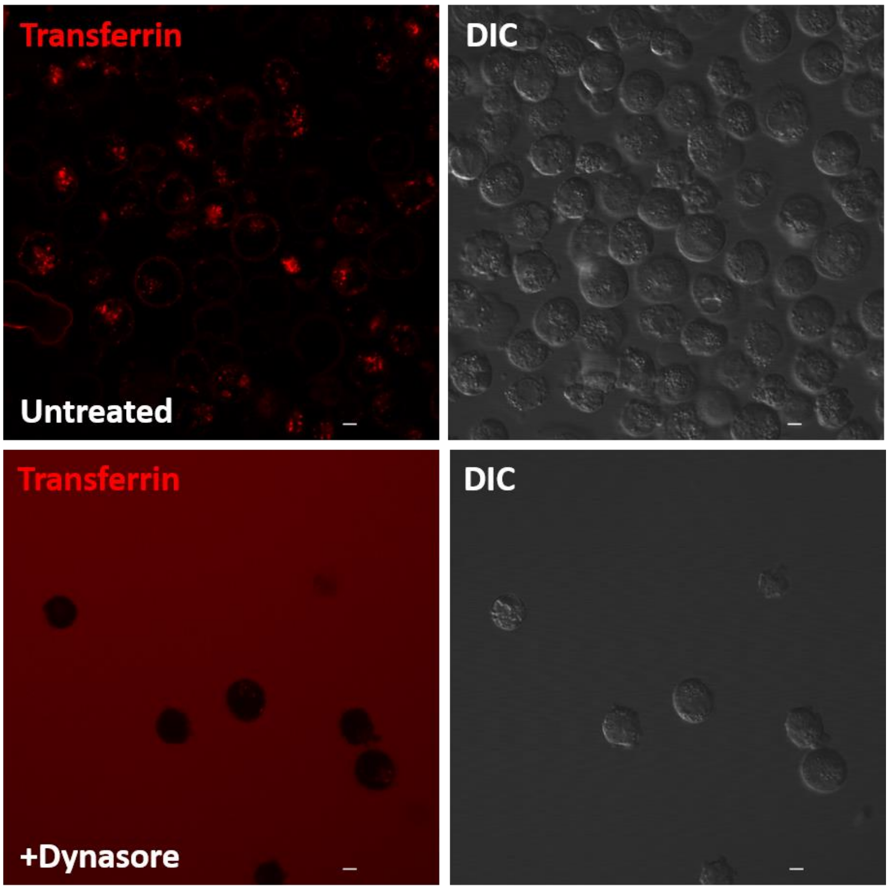

Figure S2. Effect of Dynasore on transferrin uptake by Jn.9 cells. Jn.9 cells were treated with 10 µM Dynasore for 20 min at 37 °C. One µM transferrin conjugated with AlexaFluor™ 555 (red) was added to Jn.9 cells pretreated with 10 µM Dynasore and untreated control cells. Transferrin uptake was monitored by confocal live imaging. The images presented were taken 10 min after fluorescent transferrin addition to the cells. Representative images are shown. Scale bars = 2µm.

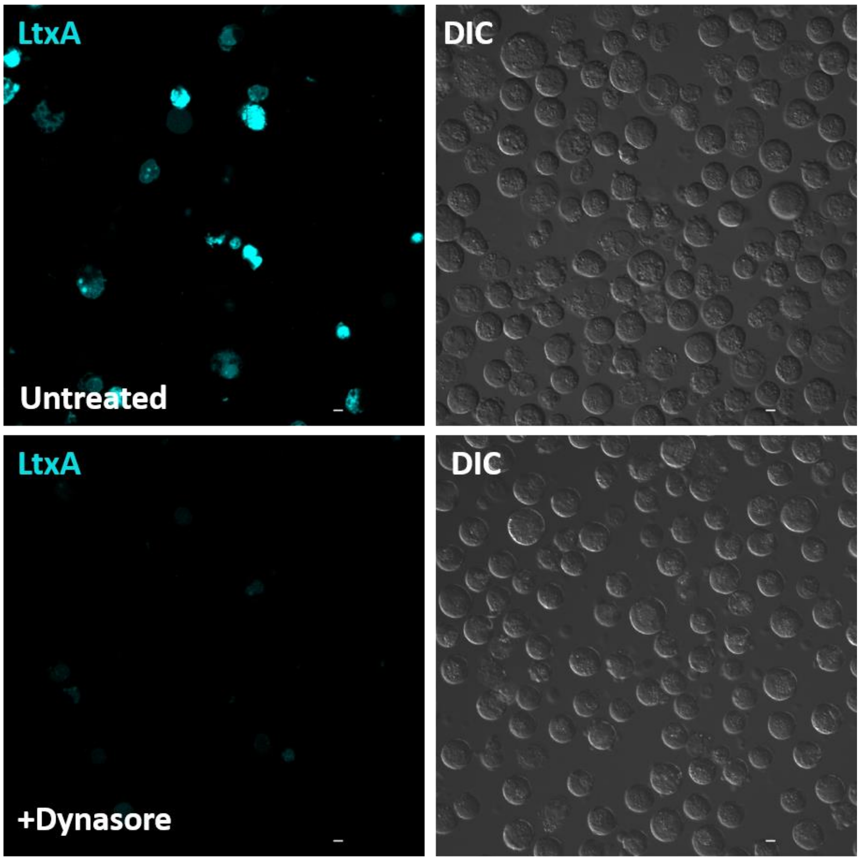

Figure S3. Effect of Dynasore on LtxA uptake by Jn.9 cells. Jn.9 cells were treated with 10 µM Dynasore for 20 min at 37 °C, and then 20 nM LtxA-DY650 (cyan) was added to the cells pretreated with Dynasore and untreated control cells. LtxA-DY650 uptake was monitored by confocal live imaging. The images presented were taken 30 min after LtxA-DY650 addition to the cells. Representative images are shown. Scale bars = 2µm.

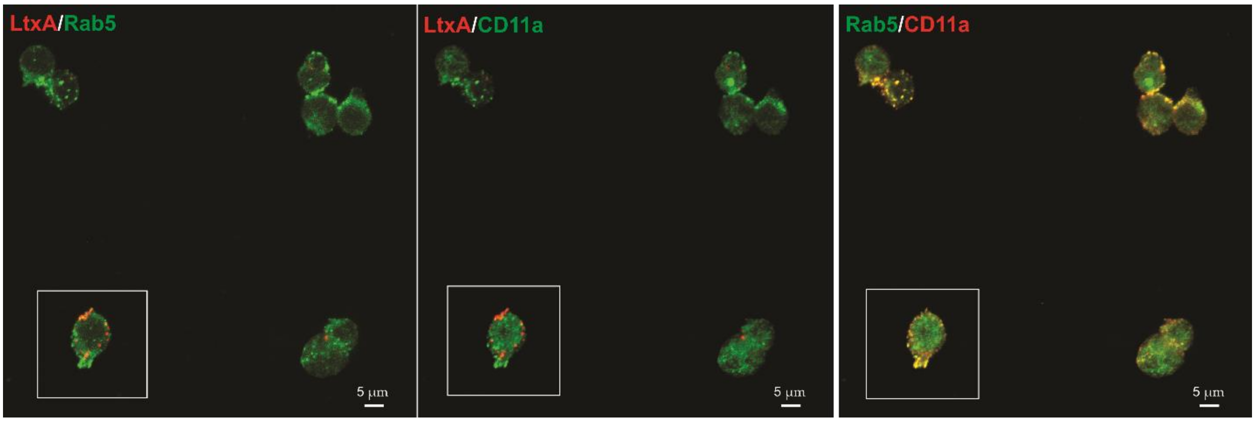

Figure S4. Localization of LtxA in early endosomes of Jn.9 cells. The cells were treated with 20 nM LtxA-DY650 for 30 min at 37 °C. Immunostaining and confocal imaging was performed as described in the *"Immunofluorescence"* section of "Material and Methods". LtxA is pseudo colored in red and CD11a is in red or green (pseudo colored), as indicated on images. Localization of LtxA-DY650 (cyan), CD11a recognized with mouse anti-human CD11a antibody conjugated with Alexa Fluor™ 594 clone HI111 (red) and Rab5 recognized by rabbit anti-Rab5 antibody followed by staining with anti-rabbit IgG Alexa Fluor®488 (green). Boxed area was used for colocalization analysis and is presented in Figure 3. Representative images are shown.

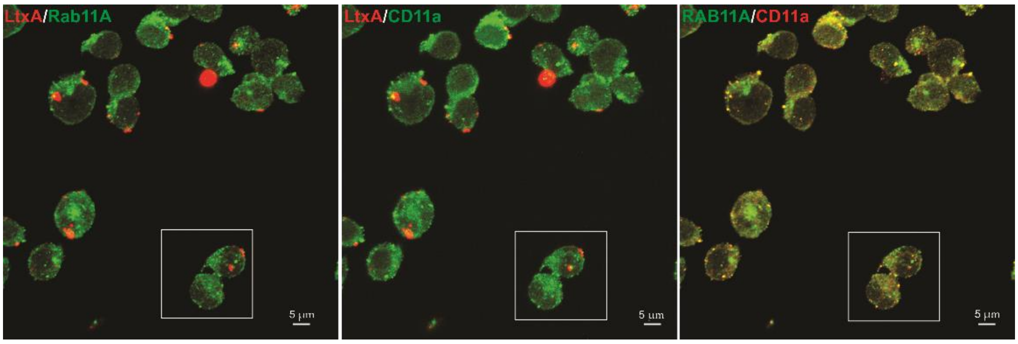

Figure S5. Localization of LtxA in Rab11A-positive endosomes of Jn.9 cells. The cells were treated with 20 nM LtxA for 30 min at 37 °C. Immunostaining and confocal imaging was performed as described in the *"Immunofluorescence"* section of "Material and Methods". LtxA is pseudo colored in red and CD11a is in red or green (pseudo colored), as indicated on images. Localization of LtxA-DY650 (cyan), CD11a recognized with mouse anti-human CD11a antibody conjugated with Alexa Fluor™ 594 clone HI111 (red) and Rab11A recognized by rabbit anti-Rab11A antibody followed by staining with anti-rabbit IgG Alexa Fluor®488 (green). Boxed cell is presented in Figure 4. Representative images are shown.

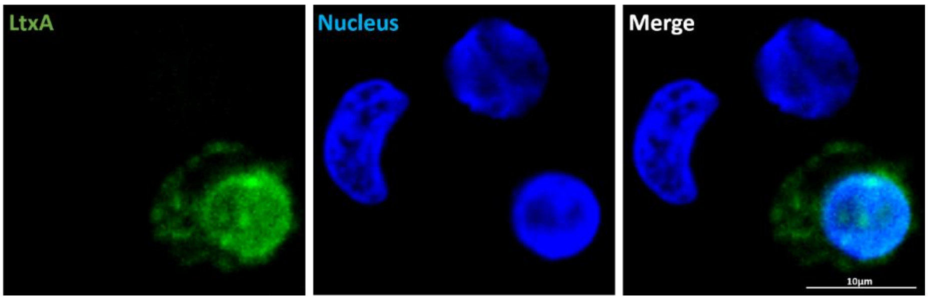

Figure S6. Localization of LtxA around nuclear membrane of Jn.9 cells. Confocal images of Jn.9 cells showing localization of LtxA-DY650 after treatment of the cells with the toxin for 2 h at 37 °C. Immunostaining and confocal imaging was performed as described in the *"Immunofluorescence"* section of "Materials and Methods". LtxA-DY650 (pseudo colored in green) and cell nuclei were stained with Hoechst 33342 (blue). Representative images are shown.

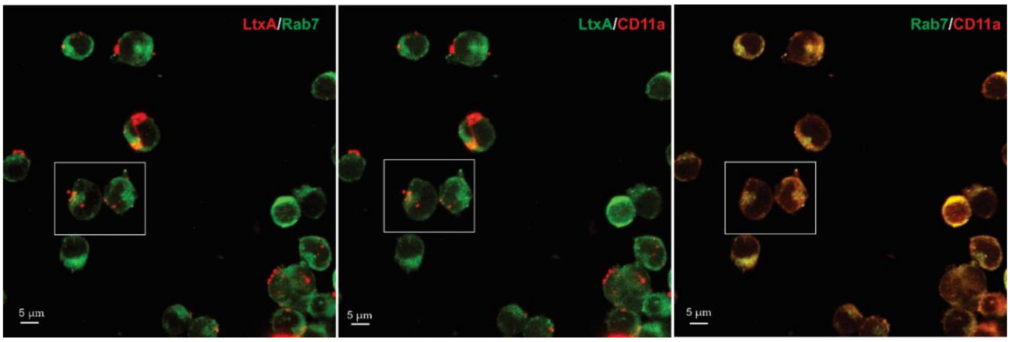

Figure 7S. Images of early endosomes in Jn.9 cells. The cells were treated with 20 nM LtxA for 30 min at 37 °C. Immunostaining and confocal imaging was performed as described in the "*Immunofluorescence*" section of "Materials and Methods". LtxA is pseudo colored in red and CD11a is in red or green (pseudo colored), as indicated on images. Localization of LtxA-DY650 (cyan), CD11a recognized with mouse anti-human CD11a antibody conjugated with Alexa Fluor™ 594 clone HI111 (red) and Rab7 recognized by rabbit anti-Rab7 antibody followed by staining with anti-rabbit IgG Alexa Fluor®488 (green). Boxed area is presented in Figure. 5. Representative cells are shown.

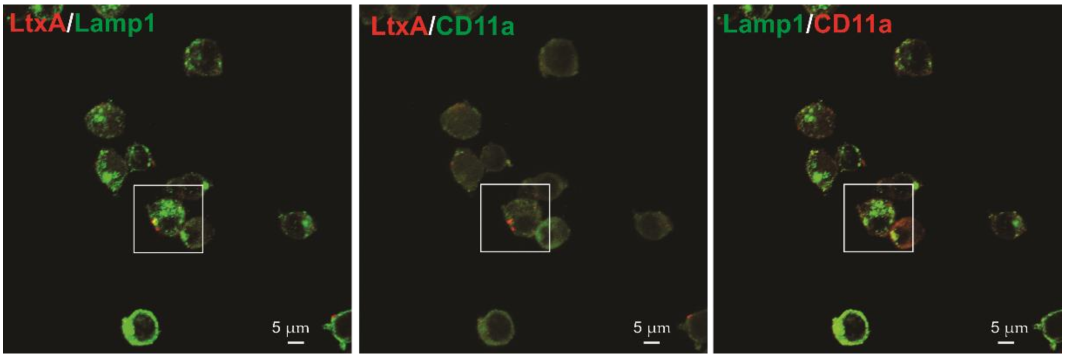

Figure 8S. Images of lysosomes in Jn.9 cells. The cells were treated with 20 nM LtxA for 30 min at 37 °C. Immunostaining and confocal imaging was performed as described in the "*Immunofluorescence*" section of "Materials and Methods". LtxA is pseudo colored in red and CD11a is in red or green (pseudo colored), as indicated on images. Localization of LtxA-DY650 (cyan), CD11a recognized with mouse anti-human CD11a antibody conjugated with Alexa Fluor™ 594 clone HI111 (red) and Lamp1 recognized with rabbit anti-Lamp1 antibody followed by staining with anti-rabbit IgG Alexa Fluor®488 (green). Boxed area is presented in Figure. 6. Representative cells are shown.

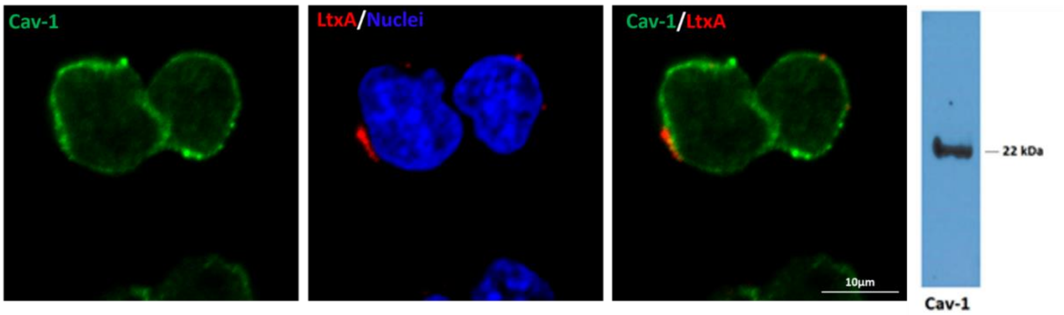

Figure 9S. Interaction of caveolin-1 and LtxA on the Jn.9 cell surface. Confocal images of colocalization of LtxA-DY650 (pseudo colored in red) and caveolin-1 (Cav-1, green) on the surface of Jn.9 cells after treatment of the cells with 20 nM LtxA-DY650 for 15 min at 37 °C. Average Pearson's coefficients for co-distribution Cav-1/LtxA is 0.8. Anti-caveolin-1 antibody from Abcam (ab32577)

were used for the experiments. Cell nuclei were stained with Hoechst 33342 (blue). The inset on the right shows Western blot analysis of caveolin-1 in Jn.9 cell lysates. Representative images are shown.

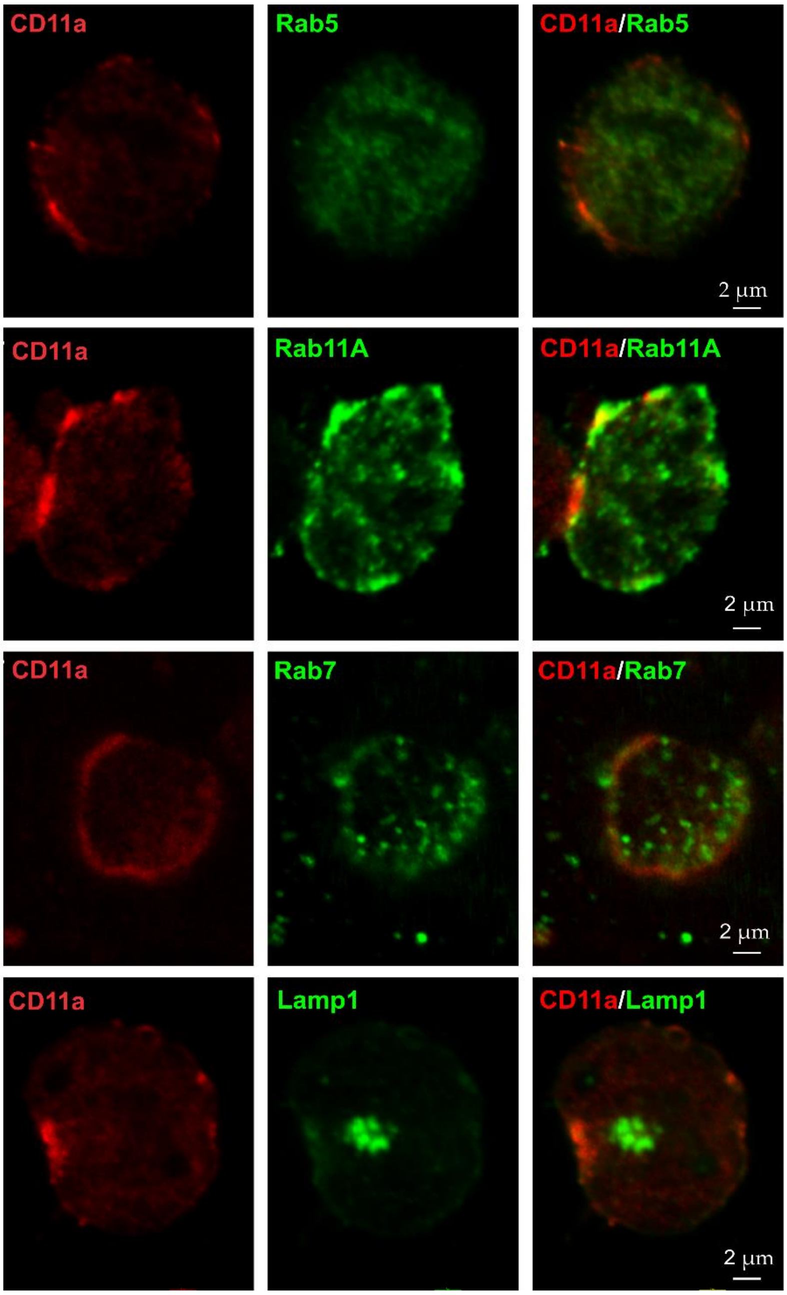

Figure 10S. Confocal imaging of CD11a trafficking in Jn.9 cells. Immunostaining and confocal imaging were performed as described in the "Immunofluorescence" section of Material and Methods. CD11a detected with mouse anti-human CD11a antibody conjugated with Alexa Fluor ™594 is shown in red, and endosomal/lysosomal markers detected through goat-anti rabbit antibody conjugated with DyLight ™ 488 are shown in green. No significant background fluorescence in unstained Jn.9 cells was detected. Representative images are shown.
